# Implementation of a maternal early warning system during early postpartum. A prospective observational study

**DOI:** 10.1371/journal.pone.0252446

**Published:** 2021-06-03

**Authors:** Cristina Ibáñez-Lorente, Rubén Casans-Francés, Soledad Bellas-Cotán, Luis E. Muñoz-Alameda

**Affiliations:** 1 Department of Anesthesia, Hospital Universitario Fundación Jiménez Díaz, Madrid, Spain; 2 Department of Anesthesia, Hospital Universitario Infanta Elena, Valdemoro, Madrid, Spain; University of Mississippi Medical Center, UNITED STATES

## Abstract

**Objective:**

To evaluate the implementation of a maternal early warning system (MEWS) for monitoring patients during the first two hours after delivery in a tertiary level hospital.

**Methods:**

Implementation of the criteria between 15 March and 15 September 2018 was evaluated in 1166 patients. The parameters collected were systolic and diastolic blood pressure, heart rate, oxygen saturation, urine output, uterine involution, and bleeding. Out-of-range values of any of these parameters triggered a warning, and an obstetrician was called to examine the patient. The obstetrician then decided whether to call the anesthesiologist. We carried out a sensitivity-specificity study of triggers and a multivariate analysis of the factors involved in developing potentially fatal disorders (PFD), reintervention, critical care admission, and stay.

**Results:**

The MEWS was triggered in 75 patients (6.43%). Leading trigger was altered systolic blood pressure in 32 patients (42.7%), and 11 patients had a PFD. Twenty-eight triggers were false-negatives. Sensitivity and specificity of the system was 0.28 (0.15, 0.45) and 0.94 (0.93, 0.96), respectively. The multivariate analysis showed a correlation between trigger activation and PFD.

**Conclusion:**

Our MEWS presented low sensitivity and high specificity, with a significant number of false-negatives.

## Introduction

Reducing morbidity and mortality in the obstetric patient continues to be a quality-of-care criterion for healthcare centers. The evolution of maternal and child mortality in recent years indicates a marked improvement in this population group [1]. Although mortality rates are very low for developed countries, the impact is high, in terms of both social repercussions and years of life lost.

One of the approaches to reduce maternal morbidity and mortality involves the use of tools to rapidly identify the patients that would benefit most from an aggressive intervention or a higher level of care. In December 2007, a review of maternal mortality concluded that 40%-50% of maternal deaths in the UK are preventable. However, early warning signs were seldom recognized [2]. Since then, most UK hospitals have introduced maternal early warning systems (MEWS) as an auditable quality of care criterion [3, 4].

Unfortunately, the implementation of these systems is not universal, and their use is uncommon outside the Anglosphere. Furthermore, although MEWS have improved maternal quality of care and the detection of adverse effects, they have had no impact on reducing maternal mortality [5].

This study evaluates the usefulness and feasibility of implementing a modified MEWS in our center, and evaluates its possible relationship with the detection and identification of adverse effects.

## Methods

### Study design and setting

This study analyses the implementation of a MEWS in a tertiary level hospital (Hospital Fundación Jiménez Díaz, Madrid, Spain). The hospital caters to all medical and surgical specialties, but does not have a specific building for gynecology and obstetrics.

We designed a single-arm prospective cohort study to be conducted over the first six months after implementing the system (first day of implementation: 15th March 2018). The study was approved by our hospital’s ethics committee before the start of patient recruitment (Ethics Commitee: Comité de Ética del Hospital Fundación Jiménez Díaz. Date of approval: 12th March 2018, Code: FJD-MEOWS-17–01). This study involved human participants who gave consent in writting form. This study followed the Strengthening the Reporting of Observational Studies in Epidemiology (STROBE) reporting guideline for cohort studies [6].

### Sample size calculation

To calculate the sample size [7], we considered that our model should have a minimum sensitivity and specificity of 90%. We knew that the incidence of maternal morbidity in our setting is 5% [8]; therefore, the required sample size for a precision of 0.1, a type I error of 0.05, and a dropout rate of 10% was 692 patients. Since around 1000 deliveries were recorded between 1 September 2017 and 28 February 2018, we decided to include all patients who gave birth between 15 March and 15 September 2018 and signed the informed consent form, and excluded all those who refused to take part in the study.

### Procedures

Our MEWS protocol is based on the criteria described by Mhyre et al. [9]. We registered systolic and diastolic blood pressure, heart rate, oxygen saturation, and urine output at 10, 30, 60, 90, and 120 minutes. By consensus between anesthesia and obstetrics departments, we included uterine involution measurement (by manual exploration), and bleeding greater than 500 ml in the protocol. We defined bleeding or lack of uterine involution as obstetric causes of alarm activation. S1 File shows the data collection form.

Two months before the start of implementation, we offered clinicians from both departments, including midwives, training courses in the usefulness of the MEWS and how the system would be implemented in our hospital. Overall attendance was approximately 70%. After implementing the protocol, nurses and midwives monitored the patients using a vital signs monitor (IntelliVue MP70, Koninklijke Philips N.V., Amsterdam, The Netherlands) for two hours after giving birth or undergoing a cesarean delivery, either in the delivery room or in the postanesthesia care unit (PACU). Monitoring of heart rate and blood oxygenation was continuous, and noninvasive blood pressure was measured every five minutes. The remaining parameters were recorded according to the measurement points established in the protocol. We also collected data on previous parity, preeclampsia, and multiple births as possible risk factors. Out-of range values in any parameter in the protocol triggered a warning for the midwives to call an obstetrician, who had to assess the patient within 15 minutes. If the obstetrician was unable to resolve the situation, an anesthesiologist was called. The alarm could be triggered at any time, not only at the recording points on the data collection form. In addition, the midwife could activate the alarm in the event of a clinical situation not included in the protocol that could seriously threaten the mother’s life, such as apnea or decreased level of consciousness. At the time of activation, the midwife recorded the patient’s vital parameters and minute of alarm activation. Irrespective of the data collected, the anesthesiologists followed up all patients who had given birth until discharge to identify any possible complications occuring outside the delivery room.

### Outcomes

Following WHO recommendations [10], our primary outcome was the sensitivity and specificity of our MEWS to derect a potential fatal disorder (PFD) during the patient’s hospital stay [11]. The WHO defined a PFD as one of the following criteria: admittance to a critical care unit (CCU), surgery within two hours of delivery, or length of stay of more than seven days. As secondary outcomes, we measured the correlation between alert activation and each PFD criteria separately.

### Statistical analysis

We used R v4.0.2 (The R Foundation for Statistical Computing, Vienna, Austria) and RStudio 1.2.5033 (RStudio PBC, Boston, USA) to perform statistical analysis. We analyzed outcomes depending on alarm activation. We described discrete and continuous variables as number and percentage and median (interquartile range [IQR]), and analized their differences using the Pearson test or the Wilcoxon rank-sum tests. We calculated the sensitivity, specificity, positive and negative predictive value (PPV and NPV) of triggers for PFD and for each criterion separately, with a 95% confidence interval. We performed a multivariate logistic analysis to study the correlation between outcomes and alarm activation, preeclampsia, parity, type of delivery, and multiple births. We presented the results in forest plots showing the odds ratio with a 95% confidence interval. We used Cox regression for multivariate analysis of length of stay, plotting the results in a forest plot showing the hazard ratio with a 95% confidence interval. To avoid errors due to multiple comparisons, we calculated the respective q-value for each p-value to maintain a false discovery rate below 5% [12]. We considered comparisons in which the p-value and q-value were below.05 as being statistically significant. We provide the original study databases, the step-by-step statistical analysis, and the document in R-Markdown format in S2–S4 Files.

## Results

**Fig 1:**
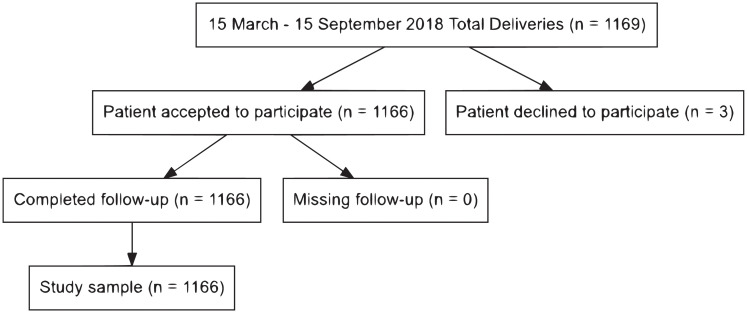
Patients flowchart according to the STROBE statement.

During the study period, there were 1169 deliveries at our hospital. Only three patients declined to participate in the study. Since there were no losses to follow-up, we included 1166 patients. Fig 1 shows the STROBE flow chart.

Median age was 34 (31–37) years, with no previous deliveries, and a rate of cesarean delivery and instrumental delivery of 23.2% and 15.1%, respectively. The protocol was triggered in 75 patients (6.43%). Patients who triggered the protocol had a higher rate of cesarean delivery (37.3% vs 22.3%, p = 0.005), preeclampsia (17.3% vs 4.8%, p < 0.001), and multiple birth (6.7% vs 1.5%). The PFD rate was also higher (14.7% vs 2.6%, p < 0.001), as was the CCU admission rate (12% vs 0.8%, p < 0.001) and length of stay [median: 2.9 (2.3–3.6) vs 2.5 (2.1–3.1) days, p = 0.005]. Fifteen patients underwent surgery in the first 2 hours after delivery, fourteen of them for uncontrolled vaginal bleeding that required obstetric curettage. One patient had to undergo emergency hysterectomy. Table 1 shows the demographic data and outcomes according to MEWS protocol activation.

**Table 1:** Demographic data and outcome results according to MEWS protocol alarm activation.

Variable	Overall (n = 1166)	No Alarm (n = 1091)	Activated Alarm (n = 75)	p-value
*Age, median* (*IQR*)	34 (31–37)	34 (31–37)	36 (29.5–39)	0.23
*Previous deliveries, n*(%)				0.63
0	782 (67.1)	730 (66.9)	52 (69.3)	
1	269 (23.1)	251 (23.0)	18 (24.0)	
≥ 2	115 (9.9)	110 (10.1)	5 (6.7)	
*Diabetes, n* (%)	99 (8.5)	90 (8.2)	9 (12.0)	0.26
*Preeclampsia, n* (%)	65 (5.6)	52 (4.8)	13 (17.3)	<0.001*
*Type of delivery, n* (%)				0.005*
Eutocic delivery	719 (61.7)	685 (62.8)	34 (45.3)	
Instrumental delivery	176 (15.1)	163 (14.9)	13 (17.3)	
Cesarean delivery	271 (23.2)	243 (22.3)	28 (37.3)	
*Multiple deliveries, n* (%)	21 (1.8)	16 (1.5)	5 (6.7)	0.009*
*Potential Fatal Disorder, n* (%)	39 (3.3)	28 (2.6)	11 (14.7)	<0.001*
*Emergency surgery within* 2 *hours, n* (%)	15 (1.3)	12 (1.1)	3 (4.0)	0.07
*Critical care admission, n* (%)	18 (1.5)	9 (0.8)	9 (12.0)	<0.001*
*Length of stay* (*h*), *median* (*IQR*)	62 (52–75)	61 (52–75)	71 (54.5–86.5)	0.005*
*Length of stay* > 7 *days, n* (%)	13 (1.1)	11 (1.0)	2 (2.7)	0.2

*: statistical significance.

The leading cause of alarm activation was altered systolic blood pressure [32 (42.7%) patients], followed by obstetric causes [24 (32%) patients]. The median time to alarm activation, obstetric assessment, refferal to an anesthesiologist, and anesthetic assessment was 10, 11, 15, and 20 minutes, respectively (Table 2). The obstetrician called the anesthesiologist in all cases. None of the calls were for life-threatening situations not included in the protocol.

**Table 2:** Causes of MEWS activation, parameter ranges, and time to assessment.

Variable	Activated Alarm (n = 75)	Lower boundary	Upper boundary
*Alarm trigger, n* (%)			
Oxygen saturation	2 (2.7)	95%	
Systolic arterial pressure	32 (42.7)	90 *mmHg*	160 *mmHg*
Diastolic arterial pressure	3 (4.0)	40 *mmHg*	100 *mmHg*
Heart rate	14 (18.7)	50 *bpm*	120 *bpm*
Obstetrics (uterine involution and bleeding)	24 (32.0)	500 *ml*	
Urine output	0 (0)	0.5 *ml*/*kg*/*h*	
*Time to activate the alarm* (*min*), *median* (*IQR*)	10 (0–60)		
*Time to obstetrics assessment* (*min*), *median* (*IQR*)	11 (2.5–60)		
*Time to call the anesthesiologist* (*min*)	15 (4.5, 61.5)		
*Time to anesthetic assessment* (*min*)	20 (7–65.5)		

Our MEWS had a sensitivity of 0.28 (0.15, 0.45), a specificity of 0.94 (0.93, 0.96), a positive predictive value of 0.15 (0.08–0.25) and a negative predictive value of 0.97 (0.96, 0.98) for PFD detection (Table 3). The protocol also showed a specificity of 0.94 and a negative predictive value of 0.99 for each of the PFD criteria separately evaluated (emergency surgery within 2 hours, CCU admission, and length of stay longer than seven days).

**Table 3:** Sensitivity, specificity, positive and negative predictive values (with 95% confidence interval) presented by the activation of our MEWS protocol alarm for the detection of PFD and its respective criteria.

Activated Alarm	Sensitivity	Specificity	Positive PV	Negative PV
*Potential fatal disorder*	0.28 (0.15, 0.45)	0.94 (0.93, 0.96)	0.15 (0.08, 0.25)	0.97 (0.96, 0.98)
*Emergency surgery within* 2 *hours*	0.20 (0.04, 0.48)	0.94 (0.92, 0.95)	0.04 (0.01, 0.11)	0.99 (0.98, 0.99)
*Critical Care Admission*	0.50 (0.26, 0.74)	0.94 (0.93, 0.96)	0.12 (0.06, 0.22)	0.99 (0.98, 1.00)
*Length of stay* > 7 *days*	0.15 (0.02, 0.45)	0.94 (0.92, 0.95)	0.03 (0.00, 0.09)	0.99 (0.98, 0.99)

PV = Predictive value.

**Fig 2:**
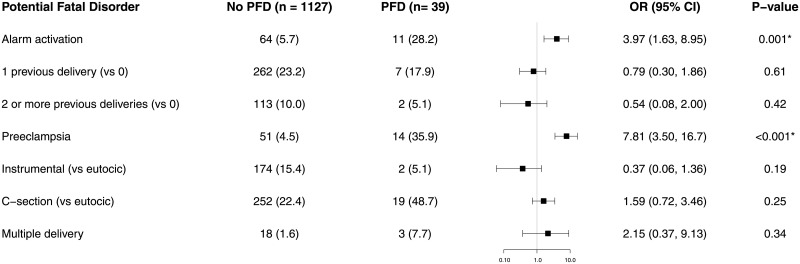
Forest plot of multivariate logistic analysis of the influence of MEWC activation, patient comorbidity and delivery on PFD. We present the results as an odds ratio with a 95% confidence interval. Results less than 1, left of the y-axis, imply risk reduction. We accepted p < 0.05 as significant.

**Fig 3:**
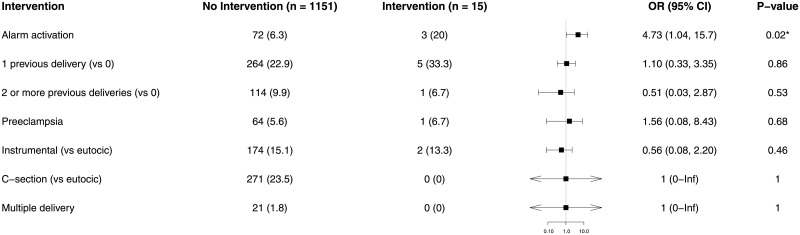
Forest plot of multivariate logistic analysis of the influence of MEWS activation, patient comorbidity and delivery on emergency surgery within two hours. We present the results as an odds ratio with a 95% confidence interval. Results less than 1, left of the y-axis, imply risk reduction. We accepted p < 0.05 as significant.

**Fig 4:**
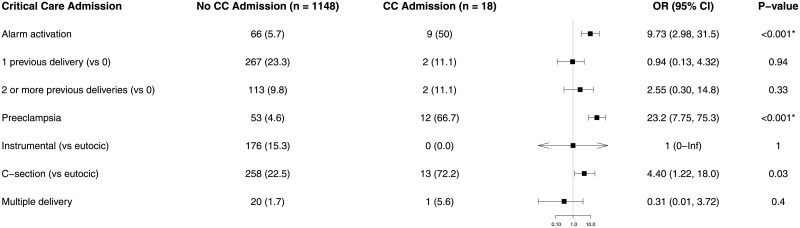
Forest plot of multivariate logistic analysis of the influence of MEWS activation, patient comorbidity and delivery on critical care unit admission. We present the results as an odds ratio with a 95% confidence interval. Results less than 1, left of the y-axis, imply risk reduction. We accepted p < 0.05 as significant.

**Fig 5:**
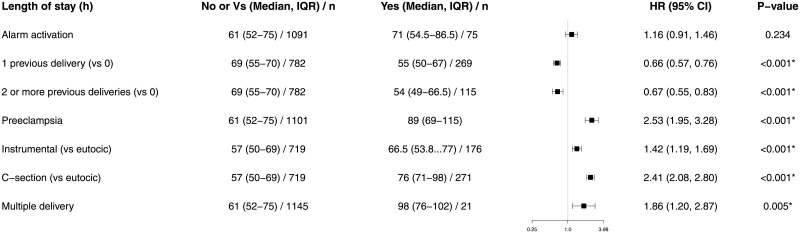
Forest plot of Cox regression of the influence of MEWS activation, patient comorbidity and delivery on length of stay. We present the results as an hazard ratio with a 95% confidence interval. Results less than 1, left of the y-axis, imply risk reduction. We accepted p < 0.05 as significant.

With regard to multivariate regression analysis, only two factors were correlated with PFD: preeclampsia [odds ratio = 7.81 (3.50, 16.7), p < 0.001] and MEWS alarm activation [odds ratio = 3.97 (1.63, 8.95), p = 0.001] (Fig 2). The only factor correlated with emergency surgery within two hours was MEWS alarm activation (odds ratio = 4.73 [1.04, 15.7], p = 0.02, Fig 3). The main factor correlated with critical care admission was preeclampsia (odds ratio = 23.2 [7.75–75.3], p < 0.001), followed by MEWS alarm activation (odds ratio = 9.73 [2.98–31.5], p < 0.001), and cesarean delivery (odds ratio = 4.40 [1.22–18], p = 0.03) (Fig 4). We found no relationship between length of stay and MEWS alarm activation in the Cox regression (Fig 5).

No significant p-values were rejected after calculating its q-value within the multiple comparability study (S3 File).

## Discussion

One of the most interesting observations arising from our study is the sensitivity, specificity, positive, and negative predictive value of MEWS alarm activation. The sensitivity is very low (0.28), while specificity and negative predictive value are very high (0.94 and 0.99, respectively). For practical purposes, these results mean that we can be confident that patients that did not trigger the MEWS protocol are unlikely to present a PFD. The negative predictive value is based on the current PFD rate in our hospital; if this were to increase, it could decrease the negative predictive value of our protocol.

Unfortunately, our low sensitivity and positive predictive value was due to the failure of the MEWS to detect many patients who developed PFD [28 (71.1%) of 39 cases]. Although we do not know the cause of such low sensitivity levels, we suspect that it is probably related to the monitoring period or the parameters included in the protocol.

In our study, patients were monitored for only 2 hours after delivery. However, monitoring was longer in studies with better sensitivity results. Singh et al. [13] published the first validation of MEWS in a prospective observational study in 676 obstetric patients to detect morbidity between the 20th week of gestation and six months postpartum. In total, 30% of the patients presented MEWS parameter alterations. They reported a MEWS sensitivity of 0.98 and a specificity of 0.79. Such an extended period of obstetric monitoring is probably impossible in our center. Given our hospital’s structure, our patients are transferred from the delivery room to a conventional ward where monitoring is not comparable to that of a PACU. Nevertheless, we suspect that extending the monitoring period from admission to 24 hours after delivery would reduce the number of false negatives.

The addition of obstetric parameters could also have modified the sensitivity and specificity of the protocol. According to Mhyre et al. [9], the simpler the warning criteria, the shorter the time needed to identify, diagnose, and treat patients with a higher risk of developing obstetric complications. Simple measures are more reliable, less vulnerable to human calculation errors, and more reproducible. Mhyre et al. [9] strongly recommend creating multidisciplinary teams that define warning criteria for each center and reviewing the evidence collected using these criteria. In 2015, Edwards et al. [14] compared the predictive value of six different MEWS to identify severe sepsis in women with chorioamnionitis and reported a sensitivity of 0.4 to 1 and a specificity of 0.04 to 0.97. The authors concluded that simple MEWS tools tended to be more sensitive, while more complex ones tended to be more specific. The addition of obstetric parameters might have complicated the protocol and thus increased specificity at the expense of lower sensitivity. It is important to bear in mind that 32% of triggers in our protocol were due to obstetric causes. Since the midwives were responsible for monitoring patients during the first two hours after delivery, we believe they might have been more selective in their notifications, and erred on notifying only for adverse obstetric situations. This could have led to an increase in specificity and decrease in sensitivity.

A high number of false positives due to an over-complex MEWS protocol is a severe limitation, a high number of false alarms can lead to professional fatigue [15]. Of course, no MEWS protocol is ideal, and we fully agree with Friedman et al. [15], that since each hospital has a unique structure, each should develop its own protocol and improve it after reviewing outcomes. We are currently in that phase—the improvement phase. Our study results encourage us to persevere in perfecting the protocol, either by increasing the monitoring time or by changing the parameters to be monitored. However, further studies are necessary to validate and generalize MEWS principles.

Finally, we would like to point out the importance of systolic blood pressure as the main trigger for our MEWS. Alterations in systolic blood pressure triggered up to 43% of cases. These activations may be related to preeclampsia. Preeclampsia was the only factor associated with PFD and critical care admission other than alarm activation in our MEWS. Thus, systolic pressure elevations in the immediate postpartum period should alert us to a possible worsening of the patient.

## Conclusion

Our MEWS protocol presents low sensitivity and high specificity with a high negative predictive value, and many false negatives. Our study shows an association between alarm activation and PFD, reintervention rate, and critical care admission rate, with preeclampsia being the main correlated factor in PFD and critical care admission.

## Supporting information

S1 File Data collection form. (DOCX)

S2 File Original study databases. (ZIP)

S3 File Statistical analysis. (HTML)

S4 File Statistical analysis raw R code. (RMD)

## References

[1] de Miguel Sesmero JR, Cacho PM, Solano AM, Feu JMO, Gómez MG, Prieto AP, et al. Mortalidad materna en España en el periodo 2010-2012: resultados de la encuesta de la Sociedad Española de Ginecología (SEGO). Progresos de Obstetricia y Ginecología. 2015.

[2] McClure JH, Cooper GM, Clutton-Brock TH, Centre for Maternal and Child Enquiries. Saving mothers’ lives: reviewing maternal deaths to make motherhood safer: 2006-8: a review. British Journal of Anaesthesia. 2011;107(2):127–132. doi: 10.1093/bja/aer192 21757549

[3] Bogod D, Mushambi M, Craig SK. Guidelines for the provision of anaesthesia services for an obstetric population. London, Royal College of Anaesthetists. 2019.

[4] Armitage M, Eddleston J, Stokes T, Guideline Development Group at the NICE. Recognising and responding to acute illness in adults in hospital: summary of NICE guidance. BMJ (Clinical research ed). 2007;335(7613):258–259. doi: 10.1136/bmj.39272.679688.47 17673769 PMC1939787

[5] Umar A, Ameh CA, Muriithi F, Mathai M. Early warning systems in obstetrics: A systematic literature review. PloS One. 2019;14(5):e0217864. doi: 10.1371/journal.pone.0217864 31150513 PMC6544303

[6] von Elm E, Altman DG, Egger M, Pocock SJ, Gøtzsche PC, Vandenbroucke JP. The Strengthening the Reporting of Observational Studies in Epidemiology (STROBE) Statement: Guidelines for reporting observational studies. International Journal of Surgery. 2014;12(12):1495–1499. doi: 10.1016/j.ijsu.2014.07.013 25046131

[7] Buderer NM. Statistical methodology: I. Incorporating the prevalence of disease into the sample size calculation for sensitivity and specificity. Academic Emergency Medicine: Official Journal of the Society for Academic Emergency Medicine. 1996;3(9):895–900. doi: 10.1111/j.1553-2712.1996.tb03538.x 8870764

[8] Cueto Hernández I. Análisis de la mortalidad y morbilidad materna según criterios de la organización mundial de la salud y del Euro-Peristat en el período 2011-2015 en el Hospital General Universitario Gregorio Marañón [info:eu-repo/semantics/doctoralThesis]. Universidad Complutense de Madrid. Madrid, España; 2018. Available from: https://eprints.ucm.es/47109/.

[9] Mhyre JM, D’Oria R, Hameed AB, Lappen JR, Holley SL, Hunter SK, et al. The maternal early warning criteria: a proposal from the national partnership for maternal safety. Obstetrics and Gynecology. 2014;124(4):782–786. doi: 10.1097/AOG.0000000000000480 25198266

[10] Say L, Souza JP, Pattinson RC, WHO working group on Maternal Mortality and Morbidity classifications. Maternal near miss–towards a standard tool for monitoring quality of maternal health care. Best Practice & Research Clinical Obstetrics & Gynaecology. 2009;23(3):287–296. doi: 10.1016/j.bpobgyn.2009.01.007 19303368

[11] Behling DJ, Renaud M. Development of an obstetric vital sign alert to improve outcomes in acute care obstetrics. Nursing for Women’s Health. 2015;19(2):128–141. doi: 10.1111/1751-486X.12185 25900584

[12] Storey JD. A direct approach to false discovery rates. Journal of the Royal Statistical Society: Series B (Statistical Methodology). 2002;64(3):479–498. doi: 10.1111/1467-9868.00346

[13] Singh S, McGlennan A, England A, Simons R. A validation study of the CEMACH recommended modified early obstetric warning system (MEOWS). Anaesthesia. 2012;67(1):12–18. doi: 10.1111/j.1365-2044.2011.06896.x 22066604

[14] Edwards SE, Grobman WA, Lappen JR, Winter C, Fox R, Lenguerrand E, et al. Modified obstetric early warning scoring systems (MOEWS): validating the diagnostic performance for severe sepsis in women with chorioamnionitis. American Journal of Obstetrics and Gynecology. 2015;212(4):536.e1–8. doi: 10.1016/j.ajog.2014.11.007 25446705

[15] Friedman AM. Maternal early warning systems. Obstetrics and Gynecology Clinics of North America. 2015;42(2):289–298. doi: 10.1016/j.ogc.2015.01.006 26002167

