# Supplementary material for: Implementation of a maternal early warning system during early postpartum. A prospective observational study
Source: PLoS One. 2021 Jun 3;16(6):e0252446. doi: 10.1371/journal.pone.0252446 (PMC8174734; doi:10.1371/journal.pone.0252446)
Supplement: S1 File — (DOCX) [file pone.0252446.s001.docx]

DATA COLLECTION FORM

**MATERNAL EARLY WARNING CRITERIA**

|  | **10 min** | **30 min** | **60 min** | **90 min** | **120 min** |
| --- | --- | --- | --- | --- | --- |
| **Sistolic Blood Pressure** |  |  |  |  |  |
| **Diastolic Blood Pressure** |  |  |  |  |  |
| **Heart Rate** |  |  |  |  |  |
| **SpO2** |  |  |  |  |  |
| **DIURESIS SI SV** | **-** | **-** | **-** | **-** |  |
| **Bleeding** |  |  |  |  |  |
| **Uterine Involution** |  | **-** |  | **-** |  |

**COMPLICATIONS S DURING DELIVERY:** YES NO

If yes, ¿which? ____________________

|  | **Call time** | **Arrival time** |
| --- | --- | --- |
| **1.Obstetrics** |  |  |
| **2. Anaesthesia** |  |  |

**WARNINGS:**

**CALL CRITERIA:**

**SBP (mmHg) < 90 o > 160**

**DBP (mmHg) > 100**

**HR < 50 o > 120**

**SpO2 basal < 95%**

**Oliguria (mL > 2h) < 35 * only if under urinary catheterization**

**Bleeding >500 within 2 hours ( If uterine atony during delivery, review as scheduled)**

**FI ATONIA EN EL PARTO REVISAR SEGÚN HORARIO DESCRITO)**
