# Supplementary material for: Implementation of a maternal early warning system during early postpartum. A prospective observational study
Source: PLoS One. 2021 Jun 3;16(6):e0252446. doi: 10.1371/journal.pone.0252446 (PMC8174734; doi:10.1371/journal.pone.0252446)
Supplement: S3 File — (HTML) [file pone.0252446.s003.html]

Statistical Analysis (step by step)


# Statistical Analysis (step by step)

#### Rubén Casans-Francés

#### 11/12/2020

This file describes how we performed the statistical analysis. There is also an R-Markdown version where you can modify or reuse the code as you wish. In case of doubt, please contact us at ruben@casans.net.

First we load the necessary libraries.

```
## 
## Attaching package: 'dplyr'
```

```
## The following objects are masked from 'package:stats':
## 
##     filter, lag
```

```
## The following objects are masked from 'package:base':
## 
##     intersect, setdiff, setequal, union
```

```
## Loading required package: ggplot2
```

```
## Loading required package: ggpubr
```

```
## 
## Attaching package: 'gridExtra'
```

```
## The following object is masked from 'package:dplyr':
## 
##     combine
```

```
## Loading required package: foreign
```

```
## Loading required package: MASS
```

```
## 
## Attaching package: 'MASS'
```

```
## The following object is masked from 'package:dplyr':
## 
##     select
```

```
## Loading required package: nnet
```

```
## 
## Attaching package: 'epiDisplay'
```

```
## The following object is masked from 'package:ggplot2':
## 
##     alpha
```

```
## 
## Attaching package: 'jtools'
```

```
## The following object is masked from 'package:epiDisplay':
## 
##     summ
```

```
## Registered S3 methods overwritten by 'lme4':
##   method                          from
##   cooks.distance.influence.merMod car 
##   influence.merMod                car 
##   dfbeta.influence.merMod         car 
##   dfbetas.influence.merMod        car
```

```
## Install package "strengejacke" from GitHub (`devtools::install_github("strengejacke/strengejacke")`) to load all sj-packages at once!
```

```
## 
## Attaching package: 'sjmisc'
```

```
## The following objects are masked from 'package:jtools':
## 
##     %nin%, center
```

```
## 
## Attaching package: 'sjlabelled'
```

```
## The following objects are masked from 'package:sjmisc':
## 
##     to_character, to_factor, to_label, to_numeric
```

```
## The following object is masked from 'package:dplyr':
## 
##     as_label
```

```
## Loading required package: knitr
```

```
## Loading required package: grid
```

```
## Loading required package: magrittr
```

```
## Loading required package: checkmate
```

We have the dataset distributed in 3 tables. We have to merge them and factor in some variables.

```
tabla<-read_ods(path="/home/rbn/Documentos/cristina/estancia.ods")
gemelar<-read_ods(path="/home/rbn/Documentos/cristina/gemelar.ods")
tabla1<-merge(tabla, gemelar, by="NumeroHistoria", all=TRUE)
tabla1$Gemelar[is.na(tabla1$Gemelar)]<-"No"
tabla1$Parto <- factor(tabla1$Parto, levels=c("Eutocic", "Instrumental","C-Section"))
tabla1$ParidadP <- factor(tabla1$ParidadP, levels=c("0", "1","≥2"))
tabla2<-read_ods(path="/home/rbn/Documentos/cristina/alerta.ods")
tabla2$act<-1
tablaunif<-subset(tabla1, Grupo == 2)
tabla3<-merge(tablaunif, tabla2, by="NumeroHistoria", all=TRUE)
tabla3<-as.data.frame(tabla3)
tabla3$act[is.na(tabla3$act)]<-0
tabla3$Activación[tabla3$act==1]<-"Si"
tabla3$Activación[tabla3$act==0]<-"No"
tabla3$Edad<-tabla3$Edad.x
tabla3$Diabetes<-factor(tabla3$Diabetes)
tabla3$Preeclampsia<-factor(tabla3$Preeclampsia)
tabla3$Diabetes<-factor(tabla3$Diabetes)
tabla3$AlertaTAS<-factor(tabla3$AlertaTAS)
tabla3$AlertaTAD<-factor(tabla3$AlertaTAD)
tabla3$AlertaFC<-factor(tabla3$AlertaFC)
tabla3$AlertaSAT<-factor(tabla3$AlertaSAT)
tabla3$Activador<-factor(tabla3$Activador, levels=c("SAT", "TAS", "TAD", "FC", "Obstetrico"))
tabla3$Diuresis500<-factor(tabla3$Diuresis500)
tabla3$Sangrado500<-factor(tabla3$Sangrado500)
tabla3$ComplicacionesParto<-factor(tabla3$ComplicacionesParto)
tabla3$AtiendeParto<-factor(tabla3$AtiendeParto, levels = c("Ginecologo", "Residente", "Matrona"))
  summary(tabla3)
```

```
##  NumeroHistoria    fechaIngreso         FechaAlta                  
##  Min.   : 300210   Length:1166        Min.   :2018-02-03 12:18:00  
##  1st Qu.:1239199   Class :character   1st Qu.:2018-03-29 10:18:15  
##  Median :1605538   Mode  :character   Median :2018-05-23 23:18:00  
##  Mean   :1499390                      Mean   :2018-05-20 23:10:19  
##  3rd Qu.:1871677                      3rd Qu.:2018-07-12 11:03:15  
##  Max.   :2018006                      Max.   :2018-09-07 13:34:00  
##                                                                    
##  FechaNacimiento                  Paridad       ParidadP          Parto    
##  Min.   :1969-05-26 01:59:59   Min.   :0.0000   0 :782   Eutocic     :719  
##  1st Qu.:1980-05-26 14:00:00   1st Qu.:0.0000   1 :269   Instrumental:176  
##  Median :1983-05-27 14:00:00   Median :0.0000   ≥2:115   C-Section   :271  
##  Mean   :1984-01-10 09:39:34   Mean   :0.4717                              
##  3rd Qu.:1987-02-28 07:00:00   3rd Qu.:1.0000                              
##  Max.   :2000-09-24 02:00:00   Max.   :7.0000                              
##                                                                            
##  Diabetes  Preeclampsia     Uci                Rea           
##  No:1067   No:1101      Length:1166        Length:1166       
##  Si:  99   Si:  65      Class :character   Class :character  
##                         Mode  :character   Mode  :character  
##                                                              
##                                                              
##                                                              
##                                                              
##     QxObs              Estancia          Edad.x       Protocolo        
##  Length:1166        Min.   :  7.00   Min.   :17.00   Length:1166       
##  Class :character   1st Qu.: 52.00   1st Qu.:31.00   Class :character  
##  Mode  :character   Median : 62.00   Median :34.00   Mode  :character  
##                     Mean   : 68.88   Mean   :33.86                     
##                     3rd Qu.: 75.00   3rd Qu.:37.00                     
##                     Max.   :525.00   Max.   :49.00                     
##                                                                        
##   Activación            Grupo    Intervencion        Criticos      
##  Length:1166        Min.   :2   Min.   :0.00000   Min.   :0.00000  
##  Class :character   1st Qu.:2   1st Qu.:0.00000   1st Qu.:0.00000  
##  Mode  :character   Median :2   Median :0.00000   Median :0.00000  
##                     Mean   :2   Mean   :0.01286   Mean   :0.01544  
##                     3rd Qu.:2   3rd Qu.:0.00000   3rd Qu.:0.00000  
##                     Max.   :2   Max.   :1.00000   Max.   :1.00000  
##                                                                    
##    Estancia7            TPM            Gemelar           FR previos       
##  Min.   :0.00000   Min.   :0.00000   Length:1166        Length:1166       
##  1st Qu.:0.00000   1st Qu.:0.00000   Class :character   Class :character  
##  Median :0.00000   Median :0.00000   Mode  :character   Mode  :character  
##  Mean   :0.01115   Mean   :0.03345                                        
##  3rd Qu.:0.00000   3rd Qu.:0.00000                                        
##  Max.   :1.00000   Max.   :1.00000                                        
##                                                                           
##      Edad.y       FR previos        Tipo de parto          TAS010     
##  Min.   :22.00   Length:1166        Length:1166        Min.   : 80.0  
##  1st Qu.:30.00   Class :character   Class :character   1st Qu.:103.5  
##  Median :36.00   Mode  :character   Mode  :character   Median :112.0  
##  Mean   :34.88                                         Mean   :117.6  
##  3rd Qu.:39.50                                         3rd Qu.:129.5  
##  Max.   :49.00                                         Max.   :184.0  
##  NA's   :1091                                          NA's   :1091   
##      TAS030         TAS060         TAS090          TAS120      AlertaTAS  
##  Min.   : 66    Min.   : 73    Min.   : 77.0   Min.   : 85.0   No  :  35  
##  1st Qu.:101    1st Qu.:106    1st Qu.:101.0   1st Qu.:104.0   Si  :  40  
##  Median :113    Median :119    Median :116.0   Median :115.0   NA's:1091  
##  Mean   :117    Mean   :122    Mean   :119.5   Mean   :120.5              
##  3rd Qu.:132    3rd Qu.:140    3rd Qu.:132.0   3rd Qu.:132.0              
##  Max.   :181    Max.   :179    Max.   :184.0   Max.   :182.0              
##  NA's   :1091   NA's   :1091   NA's   :1091    NA's   :1091               
##    MinutoTAS          TAD010           TAD030           TAD060      
##  Min.   : 10.00   Min.   : 40.00   Min.   : 42.00   Min.   : 30.00  
##  1st Qu.: 10.00   1st Qu.: 57.50   1st Qu.: 57.50   1st Qu.: 58.00  
##  Median : 30.00   Median : 65.00   Median : 66.00   Median : 67.00  
##  Mean   : 45.53   Mean   : 68.19   Mean   : 67.56   Mean   : 67.55  
##  3rd Qu.: 82.50   3rd Qu.: 75.50   3rd Qu.: 77.00   3rd Qu.: 76.00  
##  Max.   :120.00   Max.   :145.00   Max.   :104.00   Max.   :117.00  
##  NA's   :1128     NA's   :1091     NA's   :1091     NA's   :1091    
##      TAD090          TAD120      AlertaTAD     MinutoTAD         FC010       
##  Min.   :37.00   Min.   :42.00   No  :  72   Min.   :10.00   Min.   : 54.00  
##  1st Qu.:59.00   1st Qu.:61.00   Si  :   3   1st Qu.:10.00   1st Qu.: 74.50  
##  Median :66.00   Median :68.00   NA's:1091   Median :10.00   Median : 84.00  
##  Mean   :67.11   Mean   :69.33               Mean   :26.67   Mean   : 86.12  
##  3rd Qu.:75.50   3rd Qu.:76.00               3rd Qu.:35.00   3rd Qu.: 96.50  
##  Max.   :98.00   Max.   :98.00               Max.   :60.00   Max.   :152.00  
##  NA's   :1091    NA's   :1091                NA's   :1163    NA's   :1091    
##      FC030            FC060            FC090            FC120       
##  Min.   : 49.00   Min.   : 45.00   Min.   : 46.00   Min.   : 46.00  
##  1st Qu.: 72.50   1st Qu.: 74.00   1st Qu.: 75.00   1st Qu.: 74.50  
##  Median : 85.00   Median : 88.00   Median : 85.00   Median : 85.00  
##  Mean   : 86.21   Mean   : 88.29   Mean   : 86.11   Mean   : 85.61  
##  3rd Qu.: 97.00   3rd Qu.: 98.00   3rd Qu.: 96.50   3rd Qu.: 93.50  
##  Max.   :156.00   Max.   :140.00   Max.   :160.00   Max.   :135.00  
##  NA's   :1091     NA's   :1091     NA's   :1091     NA's   :1091    
##  AlertaFC      MinutoFC             SAT010           SAT010      
##  No  :  56   Length:1166        Min.   : 95.00   Min.   : 96.00  
##  Si  :  19   Class :character   1st Qu.: 97.00   1st Qu.: 97.00  
##  NA's:1091   Mode  :character   Median : 98.00   Median : 98.00  
##                                 Mean   : 98.01   Mean   : 98.13  
##                                 3rd Qu.: 99.00   3rd Qu.: 99.00  
##                                 Max.   :100.00   Max.   :100.00  
##                                 NA's   :1091     NA's   :1091    
##      SAT030           SAT060           SAT120       AlertaSAT     MinutoSAT   
##  Min.   : 76.00   Min.   : 95.00   Min.   : 94.00   No  :  72   Min.   :120   
##  1st Qu.: 97.00   1st Qu.: 98.00   1st Qu.: 97.00   Si  :   3   1st Qu.:120   
##  Median : 99.00   Median : 99.00   Median : 98.00   NA's:1091   Median :120   
##  Mean   : 97.93   Mean   : 98.31   Mean   : 98.05               Mean   :120   
##  3rd Qu.: 99.00   3rd Qu.: 99.00   3rd Qu.: 99.00               3rd Qu.:120   
##  Max.   :100.00   Max.   :100.00   Max.   :100.00               Max.   :120   
##  NA's   :1091     NA's   :1091     NA's   :1091                 NA's   :1165  
##       Activador        Minuto       Diuresis500 Sangrado500 ComplicacionesParto
##  SAT       :   2   Min.   :  0.00   No  :   5   No  :  49   No  :  42          
##  TAS       :  32   1st Qu.:  0.00   Si  :  70   Si  :  26   Si  :  33          
##  TAD       :   3   Median : 10.00   NA's:1091   NA's:1091   NA's:1091          
##  FC        :  14   Mean   : 31.47                                              
##  Obstetrico:  24   3rd Qu.: 60.00                                              
##  NA's      :1091   Max.   :120.00                                              
##                    NA's   :1091                                                
##  Tipo complicaciones intraparto MinutoPresente MinutoObstetra  
##  Length:1166                    Min.   :0      Min.   :  0.00  
##  Class :character               1st Qu.:0      1st Qu.:  2.50  
##  Mode  :character               Median :0      Median : 11.00  
##                                 Mean   :0      Mean   : 33.59  
##                                 3rd Qu.:0      3rd Qu.: 60.00  
##                                 Max.   :0      Max.   :125.00  
##                                 NA's   :1157   NA's   :1091    
##  MinutoAvisoAnestesia MinutoAnestesia Aviso obstetricia  Llegada obstetricia
##  Min.   :  0.00       Min.   :  0.0   Length:1166        Length:1166        
##  1st Qu.:  4.50       1st Qu.:  7.0   Class :character   Class :character   
##  Median : 15.00       Median : 20.0   Mode  :character   Mode  :character   
##  Mean   : 35.75       Mean   : 38.6                                         
##  3rd Qu.: 61.50       3rd Qu.: 65.5                                         
##  Max.   :125.00       Max.   :130.0                                         
##  NA's   :1091         NA's   :1091                                          
##  Aviso Anestesia    Llegada Anestesia      AtiendeParto 
##  Length:1166        Length:1166        Ginecologo:  42  
##  Class :character   Class :character   Residente :  16  
##  Mode  :character   Mode  :character   Matrona   :  17  
##                                        NA's      :1091  
##                                                         
##                                                         
##                                                         
##  Complicaciones postparto Observaciones acerca de la paciente      act         
##  Length:1166              Length:1166                         Min.   :0.00000  
##  Class :character         Class :character                    1st Qu.:0.00000  
##  Mode  :character         Mode  :character                    Median :0.00000  
##                                                               Mean   :0.06432  
##                                                               3rd Qu.:0.00000  
##                                                               Max.   :1.00000  
##                                                                                
##       Edad      
##  Min.   :17.00  
##  1st Qu.:31.00  
##  Median :34.00  
##  Mean   :33.86  
##  3rd Qu.:37.00  
##  Max.   :49.00  
##
```

We start with PFD. Let’s see how it correlates to the activation of the alert.

| Variable | Overall (n = 1166) | 0 (n = 1127) | 1 (n = 39) | P |
| --- | --- | --- | --- | --- |
| ParidadP, n (%) |  |  |  | 0.49 |
| 0 | 782 (67.1) | 752 (66.7) | 30 (76.9) |  |
| 1 | 269 (23.1) | 262 (23.2) | 7 (17.9) |  |
| ≥2 | 115 (9.9) | 113 (10.0) | 2 (5.1) |  |
| Preeclampsia, n (%) |  |  |  | <0.001 |
| No | 1101 (94.4) | 1076 (95.5) | 25 (64.1) |  |
| Si | 65 (5.6) | 51 (4.5) | 14 (35.9) |  |
| Parto, n (%) |  |  |  | <0.001 |
| Eutocic | 719 (61.7) | 701 (62.2) | 18 (46.2) |  |
| Instrumental | 176 (15.1) | 174 (15.4) | 2 (5.1) |  |
| C-Section | 271 (23.2) | 252 (22.4) | 19 (48.7) |  |
| Gemelar, n (%) |  |  |  | 0.03 |
| No | 1145 (98.2) | 1109 (98.4) | 36 (92.3) |  |
| Si | 21 (1.8) | 18 (1.6) | 3 (7.7) |  |
| Activación, n (%) |  |  |  | <0.001 |
| No | 1091 (93.6) | 1063 (94.3) | 28 (71.8) |  |
| Si | 75 (6.4) | 64 (5.7) | 11 (28.2) |  |

Later, we do the logistic regression.

| Variable | Beta (SE) | OR (95% CI) | P |
| --- | --- | --- | --- |
| Intercept | -3.86 (0.31) | - | <0.001 |
| ParidadP |  |  |  |
| 0 (ref) | - | - | - |
| 1 | -0.24 (0.46) | 0.79 (0.30, 1.86) | 0.61 |
| ≥2 | -0.63 (0.78) | 0.54 (0.08, 2.00) | 0.42 |
| Parto |  |  |  |
| Eutocic (ref) | - | - | - |
| Instrumental | -1.00 (0.77) | 0.37 (0.06, 1.36) | 0.19 |
| C-Section | 0.46 (0.40) | 1.59 (0.72, 3.46) | 0.25 |
| Preeclampsia |  |  |  |
| No (ref) | - | - | - |
| Si | 2.06 (0.40) | 7.81 (3.50, 16.74) | <0.001 |
| Gemelar |  |  |  |
| No (ref) | - | - | - |
| Si | 0.76 (0.80) | 2.15 (0.37, 9.13) | 0.34 |
| Activación |  |  |  |
| No (ref) | - | - | - |
| Si | 1.38 (0.43) | 3.97 (1.63, 8.95) | 0.001 |

And since this representation is not very visual, we generated a forest plot. We passed the results manually to a spreadsheet, called “etiqueta”.

We repeat with intervention:

```
tabmulti(Edad + ParidadP + Diabetes + Preeclampsia + Parto + Gemelar + TPM + QxObs + Criticos + Estancia + Estancia7 ~ Activación, 
         data = tabla3,
         ymeasures= c("median", "freq", "freq", "freq", "freq", "freq", "freq", "freq" , "freq" , "median" , "freq"),
         columns=c("overall", "xgroups", "p"),
         n.headings=TRUE) %>% kable()
```

```
## Mann-Whitney U was used to test whether the distribution of Edad differs in the two groups.
```

```
## Mann-Whitney U was used to test whether the distribution of Estancia differs in the two groups.
```

| Variable | Overall (n = 1166) | No (n = 1091) | Si (n = 75) | P |
| --- | --- | --- | --- | --- |
| Edad, Median (IQR) | 34.0 (6.0) | 34.0 (6.0) | 36.0 (9.5) | 0.23 |
| ParidadP, n (%) |  |  |  | 0.63 |
| 0 | 782 (67.1) | 730 (66.9) | 52 (69.3) |  |
| 1 | 269 (23.1) | 251 (23.0) | 18 (24.0) |  |
| ≥2 | 115 (9.9) | 110 (10.1) | 5 (6.7) |  |
| Diabetes, n (%) |  |  |  | 0.26 |
| No | 1067 (91.5) | 1001 (91.8) | 66 (88.0) |  |
| Si | 99 (8.5) | 90 (8.2) | 9 (12.0) |  |
| Preeclampsia, n (%) |  |  |  | <0.001 |
| No | 1101 (94.4) | 1039 (95.2) | 62 (82.7) |  |
| Si | 65 (5.6) | 52 (4.8) | 13 (17.3) |  |
| Parto, n (%) |  |  |  | 0.005 |
| Eutocic | 719 (61.7) | 685 (62.8) | 34 (45.3) |  |
| Instrumental | 176 (15.1) | 163 (14.9) | 13 (17.3) |  |
| C-Section | 271 (23.2) | 243 (22.3) | 28 (37.3) |  |
| Gemelar, n (%) |  |  |  | 0.009 |
| No | 1145 (98.2) | 1075 (98.5) | 70 (93.3) |  |
| Si | 21 (1.8) | 16 (1.5) | 5 (6.7) |  |
| TPM, n (%) |  |  |  | <0.001 |
| 0 | 1127 (96.7) | 1063 (97.4) | 64 (85.3) |  |
| 1 | 39 (3.3) | 28 (2.6) | 11 (14.7) |  |
| QxObs, n (%) |  |  |  | 0.07 |
| No | 1151 (98.7) | 1079 (98.9) | 72 (96.0) |  |
| Si | 15 (1.3) | 12 (1.1) | 3 (4.0) |  |
| Criticos, n (%) |  |  |  | <0.001 |
| 0 | 1148 (98.5) | 1082 (99.2) | 66 (88.0) |  |
| 1 | 18 (1.5) | 9 (0.8) | 9 (12.0) |  |
| Estancia, Median (IQR) | 62.0 (23.0) | 61.0 (23.0) | 71.0 (32.0) | 0.005 |
| Estancia7, n (%) |  |  |  | 0.20 |
| 0 | 1153 (98.9) | 1080 (99.0) | 73 (97.3) |  |
| 1 | 13 (1.1) | 11 (1.0) | 2 (2.7) |  |

| Variable | Beta (SE) | OR (95% CI) | P |
| --- | --- | --- | --- |
| Intercept | -4.13 (0.39) | - | <0.001 |
| ParidadP |  |  |  |
| 0 (ref) | - | - | - |
| 1 | 0.10 (0.58) | 1.10 (0.33, 3.35) | 0.86 |
| ≥2 | -0.68 (1.07) | 0.51 (0.03, 2.87) | 0.53 |
| Parto |  |  |  |
| Eutocic (ref) | - | - | - |
| Instrumental | -0.58 (0.79) | 0.56 (0.08, 2.20) | 0.46 |
| C-Section | -16.69 (1041.99) | 0.00 (0.00, 1047588218174610.00) | 0.99 |
| Preeclampsia |  |  |  |
| No (ref) | - | - | - |
| Si | 0.45 (1.06) | 1.56 (0.08, 8.43) | 0.68 |
| Gemelar |  |  |  |
| No (ref) | - | - | - |
| Si | -15.04 (3343.47) | 0.00 (NA, 10494081488018771352603621944403720224346845022993760721503266670784438908924091745718713188352.00) | 1.00 |
| Activación |  |  |  |
| No (ref) | - | - | - |
| Si | 1.55 (0.67) | 4.73 (1.04, 15.76) | 0.02 |

| Variable | Overall (n = 1166) | 0 (n = 1151) | 1 (n = 15) | P |
| --- | --- | --- | --- | --- |
| ParidadP, n (%) |  |  |  | 0.66 |
| 0 | 782 (67.1) | 773 (67.2) | 9 (60.0) |  |
| 1 | 269 (23.1) | 264 (22.9) | 5 (33.3) |  |
| ≥2 | 115 (9.9) | 114 (9.9) | 1 (6.7) |  |
| Preeclampsia, n (%) |  |  |  | 0.58 |
| No | 1101 (94.4) | 1087 (94.4) | 14 (93.3) |  |
| Si | 65 (5.6) | 64 (5.6) | 1 (6.7) |  |
| Parto, n (%) |  |  |  | 0.05 |
| Eutocic | 719 (61.7) | 706 (61.3) | 13 (86.7) |  |
| Instrumental | 176 (15.1) | 174 (15.1) | 2 (13.3) |  |
| C-Section | 271 (23.2) | 271 (23.5) | 0 (0.0) |  |
| Gemelar, n (%) |  |  |  | 1.00 |
| No | 1145 (98.2) | 1130 (98.2) | 15 (100.0) |  |
| Si | 21 (1.8) | 21 (1.8) | 0 (0.0) |  |
| Activación, n (%) |  |  |  | 0.07 |
| No | 1091 (93.6) | 1079 (93.7) | 12 (80.0) |  |
| Si | 75 (6.4) | 72 (6.3) | 3 (20.0) |  |

With CCU admission:

| Variable | Overall (n = 1166) | 0 (n = 1148) | 1 (n = 18) | P |
| --- | --- | --- | --- | --- |
| ParidadP, n (%) |  |  |  | 0.53 |
| 0 | 782 (67.1) | 768 (66.9) | 14 (77.8) |  |
| 1 | 269 (23.1) | 267 (23.3) | 2 (11.1) |  |
| ≥2 | 115 (9.9) | 113 (9.8) | 2 (11.1) |  |
| Preeclampsia, n (%) |  |  |  | <0.001 |
| No | 1101 (94.4) | 1095 (95.4) | 6 (33.3) |  |
| Si | 65 (5.6) | 53 (4.6) | 12 (66.7) |  |
| Parto, n (%) |  |  |  | <0.001 |
| Eutocic | 719 (61.7) | 714 (62.2) | 5 (27.8) |  |
| Instrumental | 176 (15.1) | 176 (15.3) | 0 (0.0) |  |
| C-Section | 271 (23.2) | 258 (22.5) | 13 (72.2) |  |
| Gemelar, n (%) |  |  |  | 0.28 |
| No | 1145 (98.2) | 1128 (98.3) | 17 (94.4) |  |
| Si | 21 (1.8) | 20 (1.7) | 1 (5.6) |  |
| Activación, n (%) |  |  |  | <0.001 |
| No | 1091 (93.6) | 1082 (94.3) | 9 (50.0) |  |
| Si | 75 (6.4) | 66 (5.7) | 9 (50.0) |  |

| Variable | Beta (SE) | OR (95% CI) | P |
| --- | --- | --- | --- |
| Intercept | -6.15 (0.73) | - | <0.001 |
| ParidadP |  |  |  |
| 0 (ref) | - | - | - |
| 1 | -0.07 (0.85) | 0.94 (0.13, 4.32) | 0.94 |
| ≥2 | 0.94 (0.95) | 2.55 (0.30, 14.82) | 0.33 |
| Parto |  |  |  |
| Eutocic (ref) | - | - | - |
| Instrumental | -15.48 (1214.08) | 0.00 (NA, 8555869955967295021907968.00) | 0.99 |
| C-Section | 1.48 (0.68) | 4.40 (1.22, 18.05) | 0.03 |
| Preeclampsia |  |  |  |
| No (ref) | - | - | - |
| Si | 3.15 (0.57) | 23.23 (7.75, 75.38) | <0.001 |
| Gemelar |  |  |  |
| No (ref) | - | - | - |
| Si | -1.12 (1.33) | 0.33 (0.01, 3.37) | 0.40 |
| Activación |  |  |  |
| No (ref) | - | - | - |
| Si | 2.27 (0.59) | 9.73 (2.98, 31.53) | <0.001 |

And Length of stay:

| Variable | Overall (n = 1166) | 0 (n = 1153) | 1 (n = 13) | P |
| --- | --- | --- | --- | --- |
| ParidadP, n (%) |  |  |  | 0.50 |
| 0 | 782 (67.1) | 771 (66.9) | 11 (84.6) |  |
| 1 | 269 (23.1) | 267 (23.2) | 2 (15.4) |  |
| ≥2 | 115 (9.9) | 115 (10.0) | 0 (0.0) |  |
| Preeclampsia, n (%) |  |  |  | <0.001 |
| No | 1101 (94.4) | 1094 (94.9) | 7 (53.8) |  |
| Si | 65 (5.6) | 59 (5.1) | 6 (46.2) |  |
| Parto, n (%) |  |  |  | <0.001 |
| Eutocic | 719 (61.7) | 716 (62.1) | 3 (23.1) |  |
| Instrumental | 176 (15.1) | 176 (15.3) | 0 (0.0) |  |
| C-Section | 271 (23.2) | 261 (22.6) | 10 (76.9) |  |
| Gemelar, n (%) |  |  |  | 0.02 |
| No | 1145 (98.2) | 1134 (98.4) | 11 (84.6) |  |
| Si | 21 (1.8) | 19 (1.6) | 2 (15.4) |  |
| Activación, n (%) |  |  |  | 0.20 |
| No | 1091 (93.6) | 1080 (93.7) | 11 (84.6) |  |
| Si | 75 (6.4) | 73 (6.3) | 2 (15.4) |  |

Except that here we perform COX regression

```
## Call:
## coxph(formula = Surv(tabla3$Estancia) ~ tabla3$Activación + 
##     tabla3$Parto + tabla3$ParidadP + tabla3$Preeclampsia + tabla3$Gemelar)
## 
##   n= 1166, number of events= 1166 
## 
##                              coef exp(coef) se(coef)       z Pr(>|z|)    
## tabla3$ActivaciónSi      -0.14410   0.86580  0.12118  -1.189 0.234392    
## tabla3$PartoInstrumental -0.34886   0.70549  0.08873  -3.931 8.44e-05 ***
## tabla3$PartoC-Section    -0.88093   0.41440  0.07661 -11.498  < 2e-16 ***
## tabla3$ParidadP1          0.41857   1.51978  0.07507   5.575 2.47e-08 ***
## tabla3$ParidadP≥2         0.39506   1.48447  0.10378   3.807 0.000141 ***
## tabla3$PreeclampsiaSi    -0.92639   0.39598  0.13275  -6.978 2.99e-12 ***
## tabla3$GemelarSi         -0.61787   0.53909  0.22259  -2.776 0.005505 ** 
## ---
## Signif. codes:  0 '***' 0.001 '**' 0.01 '*' 0.05 '.' 0.1 ' ' 1
## 
##                          exp(coef) exp(-coef) lower .95 upper .95
## tabla3$ActivaciónSi         0.8658     1.1550    0.6828    1.0979
## tabla3$PartoInstrumental    0.7055     1.4174    0.5929    0.8395
## tabla3$PartoC-Section       0.4144     2.4131    0.3566    0.4815
## tabla3$ParidadP1            1.5198     0.6580    1.3118    1.7607
## tabla3$ParidadP≥2           1.4845     0.6736    1.2113    1.8193
## tabla3$PreeclampsiaSi       0.3960     2.5254    0.3053    0.5137
## tabla3$GemelarSi            0.5391     1.8550    0.3485    0.8339
## 
## Concordance= 0.684  (se = 0.008 )
## Likelihood ratio test= 317.5  on 7 df,   p=<2e-16
## Wald test            = 282.9  on 7 df,   p=<2e-16
## Score (logrank) test = 298  on 7 df,   p=<2e-16
```

And to facilitate the reading of the forest plot, we represent HR as inverse, so that the detrimental factor is also to the right, as in the previous graphs.

```
etiqueta<-read_ods(path="/home/rbn/Documentos/cristina/etiquetaestancia.ods", sheet = 1)
forestplot(labeltext=cbind(etiqueta$Label1, etiqueta$Label2, etiqueta$Label3, etiqueta$Label4, etiqueta$Label5),
           mean=etiqueta$mean,
           lower=etiqueta$lower,
           upper = etiqueta$upper,
           xlog=TRUE,
           clip=c(0.25, 3.98),
           boxsize=0.15,
           xticks = c(0.25, 1, 3.98),
           xticks.digits = 5,
           graph.pos = 4,
           ci.vertices = TRUE,
           graphwidth = unit(30,"mm"), 
           col= fpColors(box = "black", lines = "black",
                    summary = "black", zero = "lightgray", text = "black",
                    axes = "black", hrz_lines = "black"),
           is.summary = etiqueta$summary)
```

Finally, we have collected all the p-values in one spreadsheet: pvalues.ods. We apply FDR and q-values and we check that there are no changes.

```
tablap <- read_ods(path="/home/rbn/Documentos/cristina/pvalues.ods", sheet = 1)
summary(tablap)
```

```
##        p         
##  Min.   :0.0000  
##  1st Qu.:0.0010  
##  Median :0.1300  
##  Mean   :0.2674  
##  3rd Qu.:0.4300  
##  Max.   :1.0000
```

```
qval<-qvalue(p=tablap$p)

tablap$q<-qval$qvalues

tablap$significativo<-tablap$q<0.05

tablap$significativooriginal<-tablap$p<0.05
tablap %>% kable()
```

| p | q | significativo | significativooriginal |
| --- | --- | --- | --- |
| 0.23000 | 0.3900000 | FALSE | FALSE |
| 0.63000 | 0.7411765 | FALSE | FALSE |
| 0.26000 | 0.4000000 | FALSE | FALSE |
| 0.00100 | 0.0030769 | TRUE | TRUE |
| 0.00500 | 0.0125000 | TRUE | TRUE |
| 0.00900 | 0.0211765 | TRUE | TRUE |
| 0.00100 | 0.0030769 | TRUE | TRUE |
| 0.07000 | 0.1400000 | FALSE | FALSE |
| 0.00100 | 0.0030769 | TRUE | TRUE |
| 0.00500 | 0.0125000 | TRUE | TRUE |
| 0.20000 | 0.3636364 | FALSE | FALSE |
| 0.00100 | 0.0030769 | TRUE | TRUE |
| 0.61000 | 0.7393939 | FALSE | FALSE |
| 0.42000 | 0.5600000 | FALSE | FALSE |
| 0.19000 | 0.3619048 | FALSE | FALSE |
| 0.25000 | 0.4000000 | FALSE | FALSE |
| 0.00100 | 0.0030769 | TRUE | TRUE |
| 0.34000 | 0.4857143 | FALSE | FALSE |
| 0.00100 | 0.0030769 | TRUE | TRUE |
| 0.86000 | 0.9555556 | FALSE | FALSE |
| 0.53000 | 0.6625000 | FALSE | FALSE |
| 0.46000 | 0.5935484 | FALSE | FALSE |
| 0.99000 | 1.0000000 | FALSE | FALSE |
| 0.68000 | 0.7771429 | FALSE | FALSE |
| 1.00000 | 1.0000000 | FALSE | FALSE |
| 0.02000 | 0.0444444 | TRUE | TRUE |
| 0.94000 | 1.0000000 | FALSE | FALSE |
| 0.33000 | 0.4857143 | FALSE | FALSE |
| 0.99000 | 1.0000000 | FALSE | FALSE |
| 0.03000 | 0.0631579 | FALSE | TRUE |
| 0.00100 | 0.0030769 | TRUE | TRUE |
| 0.40000 | 0.5517241 | FALSE | FALSE |
| 0.00100 | 0.0030769 | TRUE | TRUE |
| 0.23400 | 0.3900000 | FALSE | FALSE |
| 0.00008 | 0.0008000 | TRUE | TRUE |
| 0.00000 | 0.0000000 | TRUE | TRUE |
| 0.00000 | 0.0000003 | TRUE | TRUE |
| 0.00010 | 0.0008000 | TRUE | TRUE |
| 0.00000 | 0.0000000 | TRUE | TRUE |
| 0.00500 | 0.0125000 | TRUE | TRUE |
